# Effects of Zinc and Menthol-Based Diets on Co-Selection of Antibiotic Resistance among *E. coli* and *Enterococcus* spp. in Beef Cattle

**DOI:** 10.3390/ani11020259

**Published:** 2021-01-21

**Authors:** Sarah A. Murray, Raghavendra G. Amachawadi, Keri N. Norman, Sara D. Lawhon, Tiruvoor G. Nagaraja, James S. Drouillard, Harvey M. Scott

**Affiliations:** 1 Department of Veterinary Pathobiology, Texas A&M University, College Station, TX 77843, USA; smurray@cvm.tamu.edu (S.A.M.); slawhon@cvm.tamu.edu (S.D.L.); 2 Department of Clinical Sciences, Kansas State University, Manhattan, KS 66506, USA; agraghav@vet.k-state.edu; 3 Department of Veterinary Integrative Biosciences, Texas A&M University, College Station, TX 77843, USA; knorman@cvm.tamu.edu; 4 Department of Diagnostic Medicine and Pathobiology, Kansas State University, Manhattan, KS 66506, USA; tnagaraj@vet.k-state.edu; 5 Department of Animal Sciences and Industry, Kansas State University, Manhattan, KS 66506, USA; jdrouill@k-state.edu

**Keywords:** antibiotic resistance, antibiotic alternatives, heavy metals, essential oils

## Abstract

**Simple Summary:**

As antibiotic resistance increases globally, alternatives to antibiotics are increasingly being investigated as growth promoters, as well as preventive and therapeutic agents, particularly in agriculture. Equally important is the need for investigation into the effects of antibiotic alternatives on antibiotic resistance and particularly their risk for co-selection. In this study, we explored the prevalence of antibiotic-resistant *Escherichia coli* and *Enterococcus* spp. in cattle fed zinc, menthol or a combination of the two. We found that zinc supplementation was associated with higher levels of macrolide resistance observed among enterococcal isolates.

**Abstract:**

Antibiotic resistance represents a growing crisis in both human and veterinary medicine. We evaluated the use of antibiotic alternatives—heavy metals and essential oils—in beef cattle feeding, and their effects on Gram-negative and Gram-positive bacteria. In this randomized controlled field trial, we measured the impact of supplemental zinc and menthol on antibiotic resistance among commensal enteric bacteria of feeder cattle. Fecal suspensions were plated onto plain- and antibiotic-supplemented MacConkey and m-*Enterococcus* agar for quantification of total and antibiotic-resistant *Escherichia coli* and *Enterococcus* spp., respectively. Temporal effects on overall *E. coli* growth were significant (*p* < 0.05), and menthol was associated with decreased growth on tetracycline-supplemented agar. Zinc was associated with significant increases in growth on erythromycin-supplemented m-*Enterococcus* agar. Cattle fed zinc exhibited significantly higher levels of macrolide resistance among fecal enterococci isolates.

## 1. Introduction

Antibiotics have a long history of use in both animals and humans for the prevention, control and treatment of infectious diseases [1,2,3]. In the 1940s, sulfonamides were found to increase growth in chicks leading to almost seven decades of antibiotic use for growth promotion (AGP) in food animals. By 1951, a combination vitamin B_12_/low-dose chlortetracycline product was officially licensed for AGP use in food animals in the United States [4,5,6]. However, antibiotics are no longer labeled for growth promotion uses in the United States and most developed countries, prompting research into effective alternatives for therapeutic, preventive and growth promotion purposes [7,8]. Some of these alternatives include antimicrobial peptides, probiotics, heavy metals, clay minerals, egg yolk antibodies, essential oils and recombinant enzymes [9,10]. However, a review by Thacker et al. concluded that no currently licensed or approved compounds are equal to antibiotics, and most provide inconsistent results in terms of efficacy [11]. Additionally, there is great concern about the possibility of co-selection of antibiotic resistance among antibiotic alternatives [12].

While research to identify innovative alternatives to antibiotics is necessary, given the current global antibiotic resistance situation, it is equally important to explore the potential for undesired effects from those alternatives. Heavy metals, including copper and zinc, have been suggested as alternatives to antibiotics for growth promotion, as well as disease prevention. Zinc oxide (ZnO) fed at supra-nutritional levels has been shown to influence the gut microbiota of weaned piglets in a manner similar to growth-promoting antibiotics; however, differences in average daily gain have not always been shown to be statistically different between treated and untreated groups [13]. It is important to be aware of co-selection potential between tolerance/resistance to heavy metals and antibiotic resistance [14]. Specifically, heavy metal tolerance/resistance and antibiotic resistance genes are often carried on the same mobile genetic elements [12,15,16]. It has been documented that enteric bacterial populations subjected to high levels of copper in feed become more resistant to copper, which has been linked to both macrolide and glycopeptide resistance, while copper resistance has been reported in both Gram-negative and Gram-positive bacteria [17,18,19,20,21]. The link between metal resistance and antibiotic resistance, such as seen with zinc and methicillin in *Staphylococcus aureus* (MRSA), as well as with copper and erythromycin among enterococci has been reported in Norway [22] and elsewhere. The same relationship between metal resistance and antibiotic resistance was seen in Danish swine, with resistance to zinc encoded by the *czr* gene cluster associated with resistance to methicillin via the *mec*A gene [23,24]. This phenomenon is not restricted to Europe, as swine receiving supra-nutritional zinc in Kansas showed the same co-selection of MRSA in a dose–response relationship [25]. However, at this point in time, there are no studies reporting on the effects of supranutritional zinc supplemented in the feeder stage of beef cattle production in relation to phenotypic antibiotic resistance among *Escherichia coli* and *Enterococcus* spp. By studying whether zinc has a similar effect on *E. coli* and enterococci as compared to *Staphylococcus aureus*, we can determine if it could serve as a viable antibiotic alternative in beef cattle. 

Another suggested antibiotic alternative is the use of an essential oil compound such as menthol, *Origanum* (or oregano) or thymol, among many others. Essential oil compounds have been shown experimentally to be effective against both Gram-negative and Gram-positive bacteria [26]. In a study by Li et al. [27], piglets fed a combination of thymol and cinnamaldehyde had similar weight gain and feed efficiency as piglets fed antibiotics. Additionally, menthol has been shown to enhance weight gain in poultry [28]. The Cargill corporation has produced a proprietary blend of essential oils, including those derived from thyme, cinnamon and oregano, for supplementation in poultry in order to reduce antibiotic use [29]. Menthol has also been suggested as an antibiotic alternative in cattle, with equivocal results suggesting that it has no effect on total coliform counts in cattle feces and yields no increase in resistance to many antibiotics among *Escherichia coli* isolates. However, in the same study, it also was shown to yield increased prevalence of tetracycline-resistant *E. coli* after 30 days of menthol supplementation in feed [30]. While essential oils have also been suggested as alternatives to tylosin to prevent and control liver abscesses, Meyer et al. [31] found no difference in weight gain between cattle fed an essential oil blend and those fed tylosin; however, the total number of liver abscesses was reduced for steers fed tylosin, while cattle which received both essential oils and tylosin had a statistically significant increase in calculated yield grade [32]. At the time of writing there were no published studies concerning the effects of the interaction of zinc with essential oil compounds such as menthol. By studying both the independent effects of zinc and menthol on antibiotic resistance, and their interaction when used in combination, we can report on their usefulness and validity as alternatives to antibiotics while assessing potential risks associated with co-selection of antibiotic resistance.

## 2. Materials and Methods 

### 2.1. Experimental Design

A randomized controlled field trial using a 2 × 2 factorial design was conducted at the Kansas State University (KSU) Beef Cattle Research Center and approved by the KSU Institutional Animal Care and Use Committee (Animal Use Protocol #3334, 2 September 2014). In total, 80 steers were placed in individual pens, stratified by weight and then randomly assigned by weight block to a treatment group. Treatments were: supra-nutritional zinc fed at elevated feeding concentrations (300 ppm; *n* = 20 steers), menthol (fed as 0.3% of dry matter; *n* = 20 steers), a combination of supra-nutritional zinc and menthol (*n* = 20 steers) and a control group fed neither zinc nor menthol (*n* = 20 steers). Animals were allowed to acclimate to their pens for two weeks to ensure proper equilibration of enteric bacterial flora with neighboring cattle and the environment prior to the onset of the trial. 

Fecal samples were collected per rectum from each steer starting with Day 0 prior to initiating the experimental regimens and then again at Day 21. Animals were fed their respective treatment diet for three weeks, with the peak of treatment effect expected at Day 21 [33]. Samples were processed in the laboratory into a tube with 50% sterile glycerol at a 1:1 ratio of glycerol to feces. Tubes were then stored at −80 °C until later use.

### 2.2. Quantification, Isolation, Identification, Speciation

Samples from Day 0 were used as the baseline, and samples from Day 21 were considered the maximum treatment effect time period for analysis. Microbiological assays were conducted under Institutional Biosafety Committee registration #2017-049 and #2017-021 at Texas A and M University. Samples preserved with glycerol were thawed on ice and mixed thoroughly with phosphate-buffered saline (PBS) (Gibco Life Technologies, Thermo Scientific Microbiology, Oakwood Village, OH, USA) in a 1:10 dilution, using 9 mL of PBS and 1 g of feces. An aliquot of 50 µL of this dilution was spiral-plated using an EddyJet^®^ 2 Spiral Plater (Neutec Group Inc., Farmingdale, NY, USA) onto MacConkey agar (Difco, Becton Dickinson Sparks, MD, USA) for quantification of fecal coliforms; more specifically, we counted only lactose fermenting colonies and therefore presumptive *E. coli*. This dilution also was spiral plated to MacConkey agar supplemented with tetracycline (Sigma Aldrich, Merck, St. Louis, MO, USA) at 16 milligrams per liter (mg/L) as well as MacConkey agar supplemented with ceftriaxone (Sigma Aldrich, Merck, St. Louis, MO, USA) at 4 mg/L. This same dilution also was spiral-plated to m-*Enterococcus* agar (Difco) for quantification of enterococci, and to m-*Enterococcus* agar supplemented with tetracycline at 16 mg/L and to m-*Enterococcus* agar supplemented with erythromycin (Sigma Aldrich) at 8 mg/L. MacConkey agar plates were incubated at 37 °C for 18 h; in contrast, m-*Enterococcus* plates were incubated at 42 °C for 48 h. All plates were counted using the Flash & Go^®^ System (Neutec Group Inc.). 

Two random colonies from each plain (i.e., non-antibiotic) agar plate were selected and streaked to tryptic soy agar (TSA) agar with 5% sheep blood (Difco) for confirmation of species using Matrix-Assisted Laser Desorption/Ionization-Time of Flight (MALDI-TOF). Employing a single-use sterilized wooden toothpick, a single isolate of presumptive *E. coli*, or *Enterococcus* spp., was spread onto two spots of a reusable 96-spot target plate (Bruker Daltonik GmbH, Billerica, MA, USA). Once dry, one microliter (µL) of 70% formic acid was added to the first spot of each sample spot pair only for *Enterococcus* spp. (Gram-positive) isolates and to one empty spot to serve as a negative control. Formic acid was restricted in use to Gram-positive isolates, as it is unnecessary for Gram-negative bacteria such as *E. coli*. One µL of the bacterial test standard (BTS) solution (Bruker Daltonik GmbH) was applied to the first and second spots on the plate as a positive control. After drying of all spots, one µL of α-Cyano-4-hydroxycinnamic acid (HCCA) matrix solution (Bruker Daltonik GmbH, Billerica, MA, USA) was added to each spot, including all the sample spots, BTS spots, the formic acid negative control spot and an additional empty spot serving as a secondary negative control. The target plate was then transferred to the MALDI-TOF Microflex LT/SH (Bruker Daltonik GmbH) for reading, using MBT Compass v1.4 software (Bruker Daltonik GmbH). After confirmation of genus and species, these same isolates were used for phenotypic susceptibility testing.

### 2.3. Phenotypic Susceptibility Testing 

Susceptibility testing for all *Enterococcus* spp. and *E. coli* isolates was performed using broth microdilution via the Sensititre^®^ system (TREK, Thermo Scientific Microbiology, Oakwood Village, OH, USA) to determine minimum inhibitory concentrations (MIC) to arrays of antibiotics suited to Gram-negative and Gram-positive bacteria. Isolates were freshly plated to TSA with 5% sheep blood agar and incubated at 37 °C for 18–24 h. Subsequently, a bacterial dilution equivalent to a 0.5 McFarland standard was made using 11 mL of sterilized water. Next, 50 µL of the culture suspension was transferred to 11 mL of sterile Mueller-Hinton broth (TREK); finally, 50 µL of the broth culture was inoculated to each well of the U.S. National Antimicrobial Resistance Monitoring System (NARMS) Gram-positive CMV3AGPF plate for *Enterococcus* spp. and the Gram-negative CMV3AGNF plate for *E. coli* using the Sensititre^®^ automated inoculation delivery system (TREK). Antibiotics on the CMV3AGPF plate included: chloramphenicol, ciprofloxacin, daptomycin, erythromycin, gentamicin, kanamycin, lincomycin, linezolid, nitrofurantoin, penicillin, quinupristin/dalfopristin, streptomycin, tetracycline, tigecycline, tylosin and vancomycin (see range of concentrations in Table 1). Antibiotics on the CMV3AGNF plate included amoxicillin/clavulanic acid, ampicillin, azithromycin, cefoxitin, ceftiofur, ceftriaxone, chloramphenicol, ciprofloxacin, gentamicin, nalidixic acid, streptomycin, sulfisoxazole, tetracycline and trimethoprim/sulfamethoxazole (see range of concentrations in Table 2). Three positive control wells and one negative control well also were included on each plate. Plates were incubated at 37 °C for 18 h for the CMV3AGNF plate and 24 h for the CMV3AGPF plate, with *E. coli* ATCC 25922, *E. coli* ATCC 35218, *Pseudomonas aeroginosa* ATCC 27853, *Staphylococcus aureus* ATCC 29213 and *Enterococcus faecalis* ATCC 29212 serving as quality control strains run with each new serial number or batch of plates. Plates were read using a Sensititre OptiRead™ (TREK) device. The results initially were interpreted as susceptible, intermediate or resistant in accordance with the Clinical and Laboratory Standards Institute (CLSI) M100 document guidelines [34], or using the NARMS consensus breakpoints when a CLSI breakpoint was unavailable, via the SWIN software (TREK) (Table 1 and Table 2); later, intermediate results were recoded as susceptible for binary outcome statistical analyses purposes. MICs were plotted using Excel (Microsoft Office 2019, Microsoft Corporation, Redmon, WA, USA) and 95% exact confidence intervals for each proportion of resistance among isolates was calculated using Stata^®^ version 16.1 (StataCorp LLC, College Station, TX, USA) to create an integrated table of data and an illustrative figure, colloquially known as a “squashtogram”.

### 2.4. Statistical Analyses

All statistical analyses were performed using Stata^®^ v.16.1 (StataCorp LLC, College Station, TX, USA). To achieve normalized distributions, colony-forming units (CFU) derived quantities (CFU/g feces) were transformed to log base 10 (log_10_ CFU per gram of feces) for use as dependent variables in multi-level mixed effects linear regression. To determine the relative quantity of antibiotic-resistant log_10_ CFU per gram of feces to total log_10_ CFU per gram of feces, a new variable was created by subtracting the log_10_ CFU per gram of feces grown on antibiotic-supplemented agar from the log_10_ CFU per gram of feces of the corresponding plain agar plate. These differences were then also used as a dependent variable in multi-level mixed effects linear regression. A 3-way full-factorial model was constructed, factors being zinc (binary), menthol (binary) and sample day (2-level factor for Day 0 and Day 21). Full models were retained in all cases for biological reasons, regardless of the statistical significance of the interaction terms, necessitated because the treatments had not been applied before the Day 0 sampling was performed.

For statistical analysis of the phenotypic susceptibility of isolates, resistance to each antibiotic class (antibiotic class as defined by CLSI) was graphed by day and treatment group. The Gram-negative plate consisted of ten classes of antibiotics, and the Gram-positive plate consisted of 13 classes of antibiotics. Additionally, binary resistance to each class of antibiotic was summed for each isolate to create a new variable representing multi-drug resistance count (an integer variable), which also was graphed by day and treatment group. This variable was then used to determine multi-drug resistance as a binary variable (MDR, defined as resistance to ≥3 classes of antibiotics) for each isolate. A 3-way full factorial multi-level mixed effects logistic regression model was then used to determine the effect of sample day, zinc and/or menthol on the relative odds of MDR (a binary variable) for each of Gram-positive and Gram-negative bacteria. For each statistical model, marginal means were estimated and plotted by sample day 95% confidence intervals.

## 3. Results

### 3.1. Bacterial Identification

A total of 160 samples, 80 from each sample day, were plated to previously described agars. A total of 320 presumptive *E. coli* isolated from plain MacConkey agar, 160 from Day 0 and 160 from Day 21, were subjected to MALDI-TOF. From Day 0, 158 isolates (98.75%) were confirmed as *E. coli*. The two isolates that were not *E. coli* were identified as *Proteus mirabilis* and *Citrobacter sedlakii*. From Day 21, 159 isolates (99.40%) were confirmed as *E. coli*. The single non-*E. coli* isolate was identified as *Pseudomonas chlororaphis*.

A total of 320 presumptive *Enterococcus* spp. isolated from plain m-*Enterococcus* agar from Day 0 and Day 21 were subjected to MALDI-TOF. From Day 0, 95 (59.40%) enterococcal isolates were *E. faecium*, 33 (20.63%) were *E. hirae*, 17 (10.63%) were *E. mundtii*, 3 (1.88%) were *E. casseliflavus*, 2 (1.25%) were *E. durans*, 2 (1.25%) were *E. gallinarum*, 1 (0.63%) was *E. faecalis*, 1 (0.63%) was *E. thailandicus* and 1 (0.63%) was *E. avium*. Five out of the 160 (3.13%) isolates were not *Enterococcus* spp.; one was *Aerococcus viridans* and four could not be identified using MALDI-TOF. From Day 21, 81 (50.63%) of the enterococci were *E. faecium*, 30 (18.75%) were *E. hirae*, 21 (13.13%) were *E. mundtii*, 11 (6.88%) were *E. casseliflavus*, 9 (5.63%) were *E. faecalis*, 4 (2.5%) were *E. thailandicus* and 1 (0.63%) was *E. durans*. A total of three (1.88%) isolates from Day 21 were not *Enterococcus* spp.; one (0.63%) was *Aerococcus viridans*, one was *Streptococcus lutetiensis* and one isolate could not be identified using MALDI-TOF.

### 3.2. Multi-Level Mixed Effects Linear Regression Modeling of Plate Quantification

**Figure 1:**
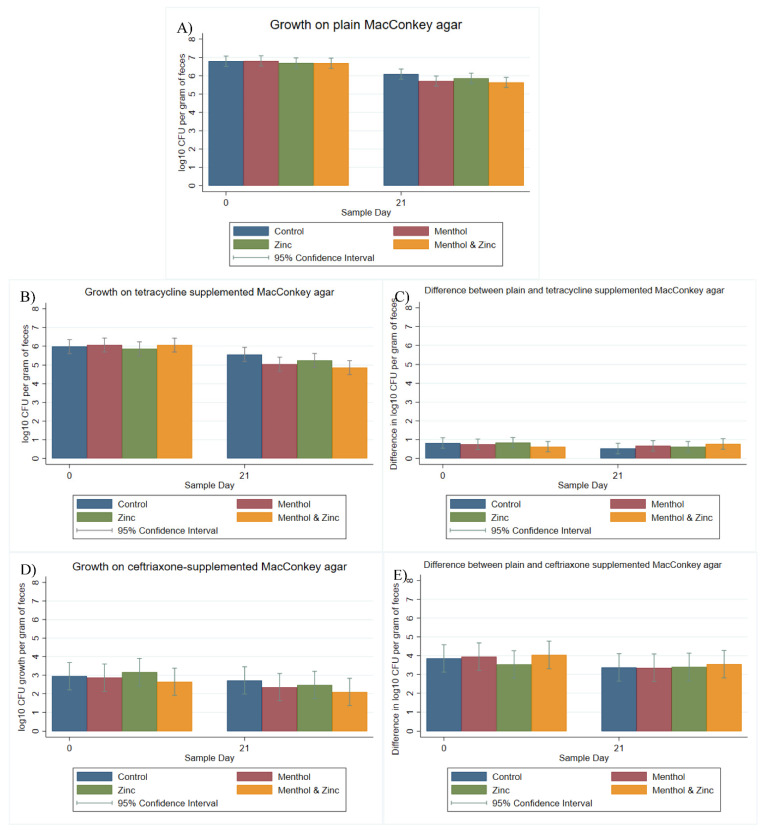
Marginal means with 95% confidence intervals of a 2 × 2 × 2 multi-level mixed linear regression model, using factors of zinc, menthol and sample day. (**A**) log_10_ colony-forming units (CFU) per gram of feces on plain MacConkey agar, (**B**) log_10_ CFU per gram of feces on tetracycline-supplemented (16 mg/L) MacConkey agar, (**C**) Difference in log_10_ CFU between plain and tetracycline-supplemented (16 mg/L) MacConkey agar, (**D**) log_10_ CFU per gram of feces on ceftriaxone-supplemented (4 mg/L) MacConkey agar and (**E**) Difference in log_10_ CFU between plain and ceftriaxone supplemented (4 mg/L) MacConkey supplemented agar.

For *E. coli* all samples (*n* = 160) were quantified (i.e., showed growth) on plain MacConkey agar, while 99.37% (*n* = 159) were quantified on tetracycline-supplemented MacConkey and 73.12% (*n* = 117) of samples were quantified on ceftriaxone-supplemented agar; for statistical analyses, samples exhibiting no growth were recorded as zero (0). Samples which were not quantified exhibited no growth (no samples were coded as too numerous to count—TNTC). The single sample which exhibited no growth on tetracycline-supplemented agar was from the combined zinc and menthol group on Day 21. For plain MacConkey agar (Figure 1A), the sampling period significantly (*p <* 0.05) affected log_10_ CFU per gram of feces, while the treatment group did not influence the outcome independent of period effects. In comparison, concerning the results of growth on tetracycline-supplemented MacConkey agar both the menthol and the combined menthol and zinc groups exhibited a statistically significant (*p* < 0.05) decrease in log_10_ CFU per gram of feces (Figure 1B) from Day 0 to Day 21.

By subtracting the log_10_ growth on tetracycline-supplemented agar from corresponding growth on plain MacConkey agar the difference is presented graphically, generally as a positive number. Using this difference in log_10_ CFU (x), expressed as 10^−x^, a difference of 1 is expressed as 10^−1^ = 0.1, a difference of 2 is expressed as 10^−2^ = 0.01, each serving as a crude estimate of the prevalence of tetracycline resistance among coliforms. Therefore, the difference in growth between plain and antibiotic supplemented agar with respect to resistance is inversely related, and a decrease in the difference should be interpreted as an increase in the level of antibiotic resistance.

When looking at the difference between growth on plain and tetracycline-supplemented MacConkey agar, neither sample day nor treatment had a significant (*p* > 0.05) effect (Figure 1C). Additionally, growth on ceftriaxone-supplemented MacConkey agar exhibited no significant effects (*p* > 0.05) for day or treatment group. The samples which did not grow on ceftriaxone-supplemented agar (coded as zero) were spread equally across treatments. There was a tendency across treatment groups for decreased log_10_ CFU from Day 0 to Day 21 on ceftriaxone-supplemented agar; however, these too were not significant (Figure 1D). Neither day nor treatment group significantly impacted the difference in log_10_ CFU per gram of feces growth on plain versus ceftriaxone-supplemented MacConkey agar (Figure 1E).

**Figure 2:**
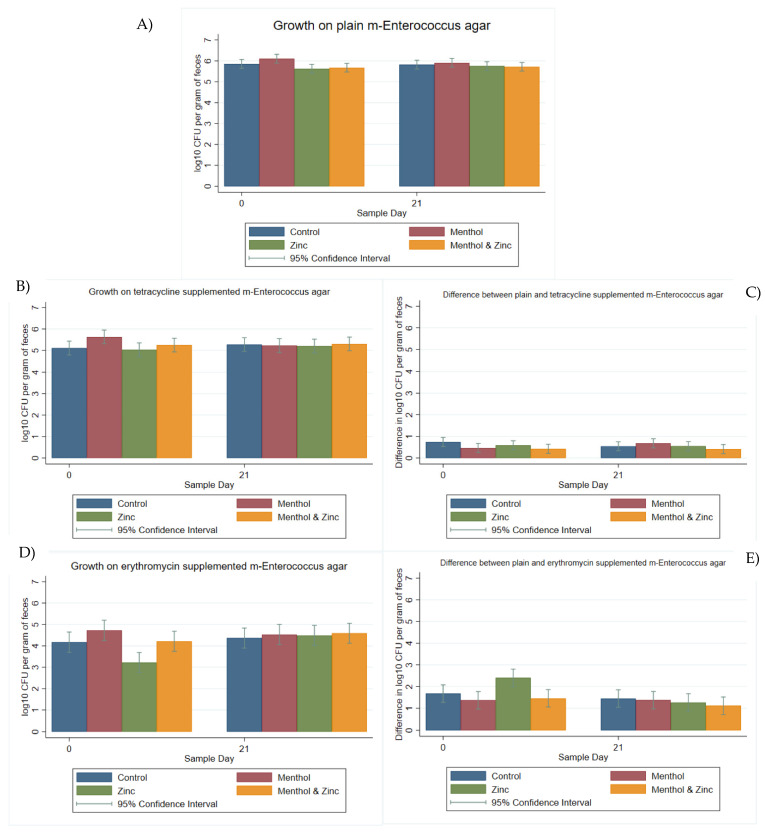
Marginal means with 95% confidence intervals of a 2 × 2 × 2 multi-level mixed linear regression model, using factors of zinc, menthol and sample day. (**A**) log_10_ CFU per gram of feces on plain m-*Enterococcus* agar, (**B**) log_10_ CFU per gram of feces on tetracycline-supplemented m-*Enterococcus* agar, (**C**) Difference in log_10_ CFU between plain and tetracycline-supplemented m-*Enterococcus* agar, (**D**) log_10_ CFU per gram of feces on erythromycin-supplemented m-*Enterococcus* agar and (**E**) Difference in log_10_ CFU between plain and erythromycin-supplemented m-*Enterococcus* agar.

For enterococci, all samples (*n* = 160) were quantifiable on both m-*Enterococcus* agar and m-*Enterococcus* agar supplemented with tetracycline, while 96.87% (*n* = 155) were quantifiable (i.e., growth > 0 log_10_ CFU with none TNTC) on m-*Enterococcus* agar supplemented with erythromycin. For growth on plain m-*Enterococcus* agar, neither treatment nor sample day had a significant effect (*p* > 0.05). The menthol group started on Day 0 at a significantly (*p* < 0.05) higher log_10_ CFU per gram of feces than the zinc and the combination zinc and menthol groups, despite the randomization process (Figure 2A). There were no significant differences (*p* > 0.05) among treatment groups by Day 21. The treatment groups at Day 21 were not significantly different than their baselines at Day 0. The menthol group tended to have higher log_10_ CFU per gram of feces grown on tetracycline-supplemented agar on Day 0; however, this was not significantly different (*p* > 0.05) from the other treatment groups (Figure 2B). By Day 21, there were still no significant differences among treatment groups, and no significant differences compared to Day 0 (*p* > 0.05).

Neither day nor treatment group significantly impacted the difference in log_10_ CFU per gram of feces growth on plain versus tetracycline-supplemented m-Enterococcus agar (Figure 2C). The zinc group was significantly (*p <* 0.05) lower in log_10_ CFU per gram of feces on erythromycin-supplemented agar compared to the other treatment groups on Day 0 (Figure 2D). This occurred despite the randomization process. It should be noted that of the five samples which exhibited no growth on m-*Enterococcus* agar with erythromycin, four samples belonged to the zinc treatment group and were collected on Day 0. There was a significant increase in log_10_ CFU per gram of feces growth on erythromycin-supplemented agar between Day 0 and Day 21 for the zinc group. However, it was not significantly different from the other treatment groups.

Similarly, the difference in log_10_ CFU per gram of feces growth between plain and erythromycin-supplemented m-*Enterococcus* agar was significantly different for the zinc group compared to the menthol and the combined zinc and menthol treatment groups. Correspondingly, the zinc group showed a significant decrease in growth difference from Day 0 to Day 21, (Figure 2E), suggesting approximately a ten-fold increase in erythromycin resistance during that 21-day period. Due to the significant difference in the zinc group on Day 0 compared to the other groups, a *post hoc* pairwise comparison using Bonferroni correction was performed, and the zinc group retained its experiment-wise significant (*p* < 0.05) decrease in growth difference from Day 0 to Day 21, confirming that an increase in erythromycin resistance occurred for this group.

### 3.3. Descriptive Statistics of Phenotypic Resistance of Isolates

For phenotypic resistance of *E. coli* isolates (Table 3), all isolates were susceptible to ciprofloxacin regardless of treatment group or sampling day. Nearly half of all isolates were resistant to tetracycline. Over 20% of isolates exhibited resistance to sulfisoxazole and streptomycin, while very few isolates (less than 1%) were resistant to amoxicillin/clavulanic acid, azithromycin, cefoxitin, ceftiofur, ceftriaxone, gentamicin, nalidixic acid or trimethoprim/sulfamethoxazole. It should be noted that a bimodal distribution of MIC values appeared for isolate susceptibility to ceftiofur with the majority being susceptible and having very low MIC values. This distribution was also present when contrasting the MICs of susceptible versus resistant *E. coli* for gentamicin and nalidixic acid.

**Figure 3:**
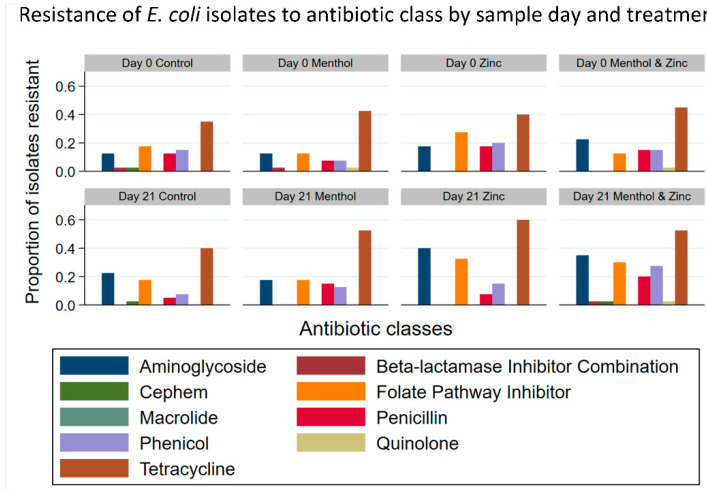
Resistance (proportion) of *Escherichia coli* isolates to each antibiotic class by sample day and treatment.

**Figure 4:**
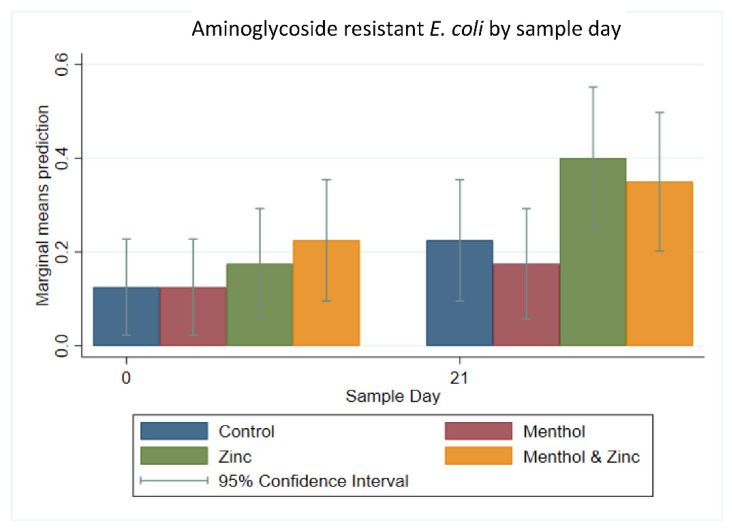
Marginal means with 95% confidence intervals of a 2 × 2 × 2 multi-level mixed logistic regression model, using factors of zinc, menthol and sample day on aminoglycoside-resistant *E. coli*.

Similarly, the resistance of *E. coli* isolates to each of the nine antibiotic classes (Figure 3) by sample day and treatment showed that aminoglycoside resistance (collapsing gentamicin and streptomycin) tended to increase from Day 0 to Day 21 across all treatment groups, from an average of 16.88% to 28.75%. This change in resistance was later tested for significance using a multi-level mixed effect logistic regression model. The predicted prevalence of aminoglycoside resistant isolates increased from Day 0 to Day 21 for the zinc group and the combined zinc and menthol group; however, this was not significant (*p* > 0.05) (Figure 4). The temporal effect of sample day alone showed an increase from Day 0 to Day 21; however, this increase was also not significant (*p* > 0.05). However, resistance to the tetracycline class tended to increase more for the menthol, zinc and combination treatment groups from Day 0 to 21 than for the control group. The menthol group increased from 42.5% isolates resistant on Day 0 to 52.5% on Day 21, the zinc group increased from 40% to 60% isolates resistant and the combined zinc and menthol group increased from 45% to 52.5% isolates resistant to tetracycline class antibiotics.

**Figure 5:**
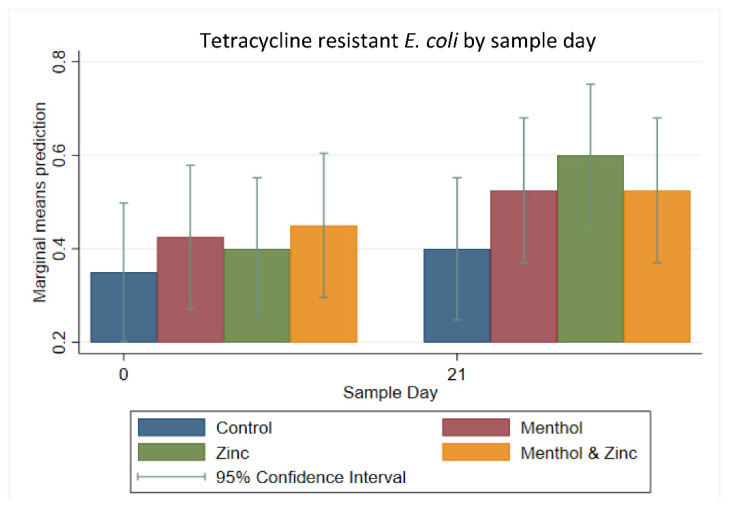
Marginal means with 95% confidence intervals of a 2 × 2 × 2 multi-level mixed logistic regression model, using factors of zinc, menthol and sample day on the binary outcome of tetracycline resistant *E. coli*.

Multi-level mixed effect logistic regression modeling was also performed on the binary outcome of resistance to tetracycline. The predicted prevalence of tetracycline-resistant *E. coli* tended to increase from Day 0 to Day 21 among all groups. Most notably, the zinc group increased in the predicted prevalence of tetracycline resistant *E. coli,* from Day 0 to Day 21; however, this increase was not significant. Additionally, sample day alone was not significant in the predicted prevalence of tetracycline resistant *E. coli* (Figure 5).

**Figure 6:**
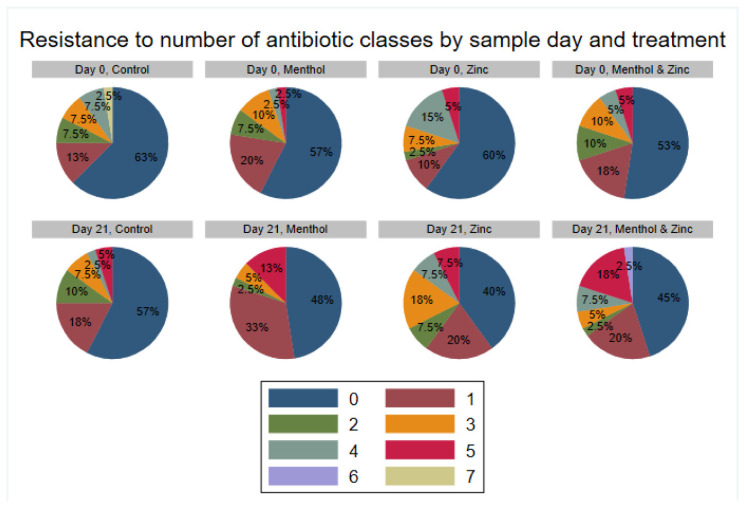
Resistance (%) of *E. coli* isolates to number of antibiotic classes by sample day and treatment. Each color indicates the specified corresponding number of antibiotic classes to which antibiotic resistance was observed.

Additionally, the resistance to the number of antibiotic classes by sample day and treatment (Figure 6) showed a trend towards increasing MDR in the zinc group from a total of 27.5% isolates MDR on Day 0 to 32.5% on Day 21. Correspondingly, the zinc group exhibited an increase in the percentage of isolates resistant to three antibiotic classes, from 7.5% at Day 0 to 18% at Day 21, and an increase in isolates resistant to five antibiotic classes, from 5% at Day 0 to 7.5% at Day 21. The menthol group did not show any trend for overall MDR, slightly increasing from 15% of isolates classified as MDR on Day 0 to 18% on Day 21. However, there was an increase in the percentage of isolates resistant to five classes of antibiotic among the menthol group, from 2.5% of isolates resistant on Day 0, to 13% on Day 21. The combined zinc and menthol group also showed an increase in percentage of MDR isolates, from 20% on Day 0 to 33% on Day 21. Correspondingly, the combined zinc and menthol group had an increase in the percent of isolates resistant to five classes of antibiotics, from 5% at Day 0 to 18% at Day 21. Additionally, the zinc and menthol group did not have any isolates resistant to six antibiotic classes on Day 0; however, on Day 21, 2.5% of isolates were resistant. Most of this gain in resistance prevalence came at the expense of the proportion of isolates that were initially pan-susceptible to all antibiotic classes on Day 0. In contrast, the control group seemingly decreased in its number of isolates resistant to multiple classes of antibiotics, with 2.5% of isolates resistant to seven classes of antibiotics and none resistant to seven classes on Day 21. These increases in MDR were statistically tested for significance using a multi-level mixed logistic regression.

**Figure 7:**
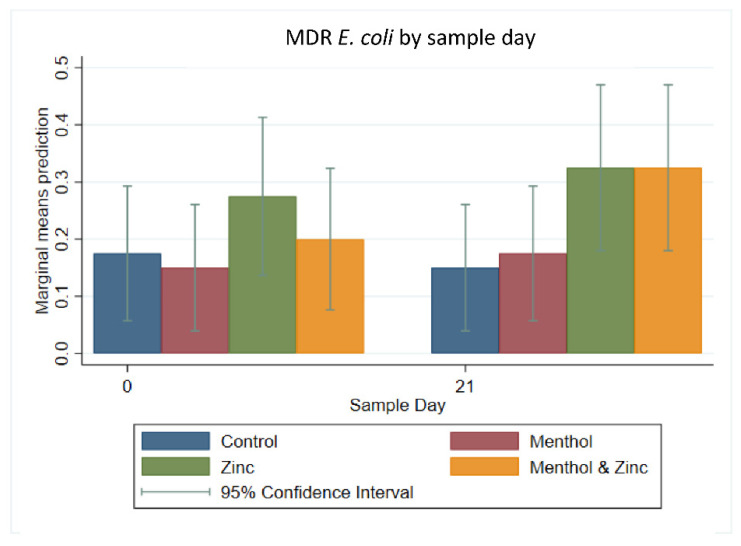
Marginal means with 95% confidence intervals of a 2 × 2 × 2 multi-level mixed logistic regression model, using factors of zinc, menthol and sample day on the binary outcome of multi-drug resistant (MDR) *E. coli*.

Multi-level mixed effect logistic regression modeling was performed on the binary outcome of MDR *E. coli* isolates (Figure 7). There were no significant differences among treatment groups. Overall, there was an increase in the predicted prevalence of MDR isolates from Day 0 to Day 21 in MDR for sample day alone; again, this was also not significant (*p* > 0.05).

For phenotypic resistance of *Enterococcus* spp. (Table 4), all isolates were susceptible to gentamicin, tigecycline and vancomycin. Nearly all isolates (91.25%) were resistant to lincomycin, while approximately a third were resistant to quinupristin/dalfopristin and tetracycline. Not surprisingly, resistance to erythromycin and tylosin (both macrolides) was nearly equal, at 17.81% and 18.44%, respectively. Less than 1% of isolates were resistant to chloramphenicol, kanamycin, linezolid, penicillin or streptomycin. It should be noted that a bimodal distribution across isolates appeared in regard to the MIC values of both tetracycline and lincomycin, corresponding to their distinct categorization as either susceptible or resistant to these two drugs.

**Figure 8:**
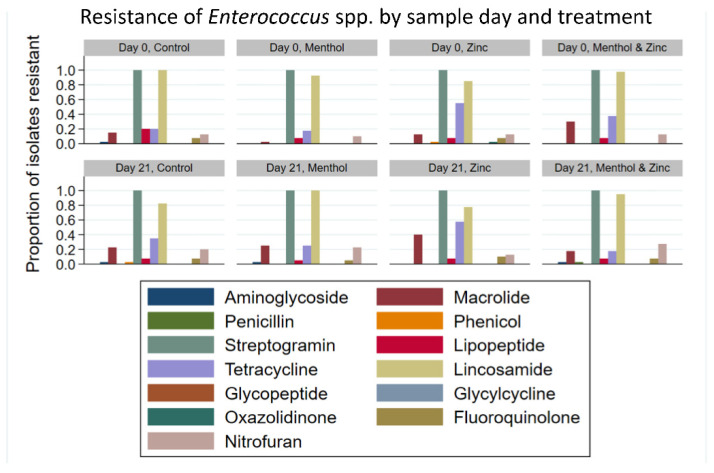
Resistance (proportion) of *Enterococcus* spp. isolates to each antibiotic class by sample day and treatment.

Additionally, analysis of the resistance of *Enterococcus* spp. isolates to each of 13 antibiotic classes by treatment group and by sample day (Figure 8), showed that tetracycline resistance tended to decrease from Day 0 to Day 21 in the menthol/zinc combination treatment group; that is, 37.5% of isolates were resistant on Day 0 compared to 17.5% of isolates resistant on Day 21. Tetracycline resistance tended to increase during the same period in the control group, from 20% of isolates on Day 0 to 35% on Day 21. Similarly, the percentage of macrolide resistant isolates tended to increase in the control group from 15% on Day 0 to 22.5% on Day 21. There was also an increased percentage of macrolide resistant isolates in both the menthol group and the zinc group, an increase from 2.5% on Day 0 to 15% on Day 21 in the menthol group, and from 12.5% to 40% on Day 21 in the zinc group. The combined zinc and menthol group tended to exhibit decreased macrolide resistance, from 30% on Day 0 to 17.5% on Day 21.

**Figure 9:**
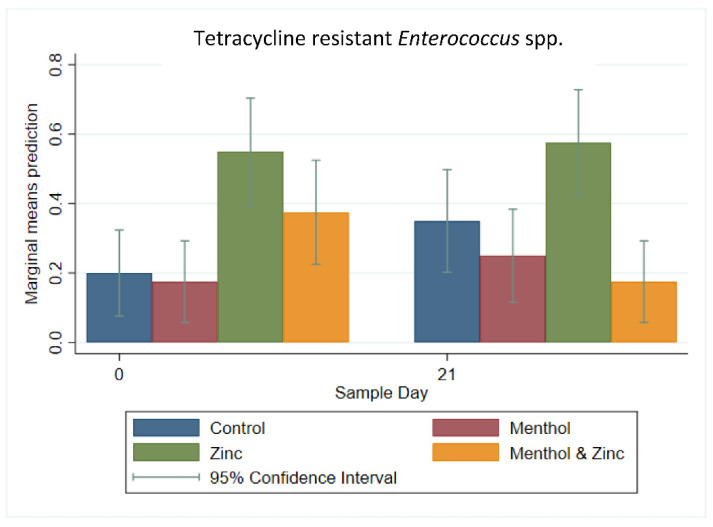
Marginal means with 95% confidence intervals of a 2 × 2 × 2 multi-level mixed logistic regression model, using factors of zinc, menthol and sample day on the binary outcome of tetracycline resistance among *Enterococcus* spp.

A multi-level logistic regression analysis for the binary outcome of tetracycline-resistance among *Enterococcus* spp. isolates showed an increase in the predicted prevalence of tetracycline resistant isolates in the control group from Day 0 to Day 21; however, this increase was not significant (*p* > 0.05). The zinc group showed a significantly (*p* < 0.05) higher predicted prevalence of tetracycline resistant isolates on Day 0, compared to the control group and the menthol group. On Day 21, the zinc group continued to show a significantly higher proportion of tetracycline-resistant isolates compared to the menthol group and the combined zinc and menthol group (Figure 9).

**Figure 10:**
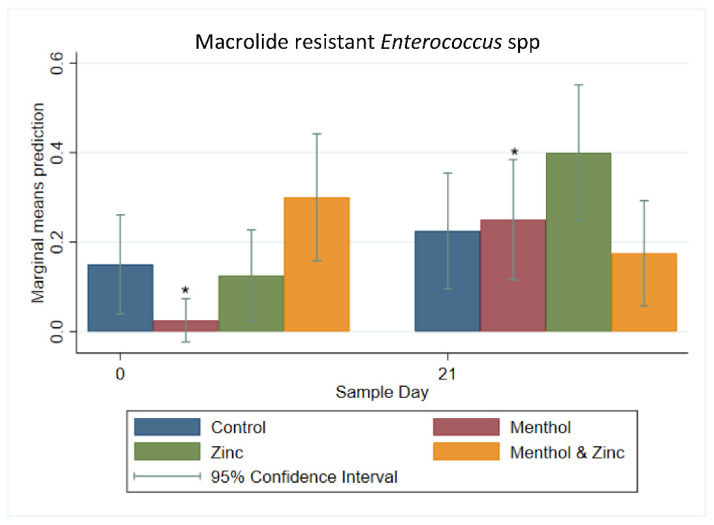
Marginal means with 95% confidence intervals of a 2 × 2 × 2 multi-level mixed logistic regression model, using factors of zinc, menthol and sample day on the binary outcome of macrolide resistant *Enterococcus* spp. * significantly different *(p* < 0.05) using a *post hoc* Bonferonni pairwise comparison.

A multi-level logistic regression on the binary outcome of macrolide-resistance for *Enterococcus* spp. isolates showed no significant differences in the predicted prevalence of macrolide resistant isolates among the treatments on Day 21 (Figure 10). Day 0 to Day 21 comparisons showed the menthol treatment and the zinc treatment with significantly increased predicted prevalence of macrolide resistant enterococci. The menthol group showed a significantly lower predicted prevalence of macrolide resistant enterococci compared to the combined menthol and zinc group on Day 0. Therefore, a *post hoc* Bonferroni multiple comparison adjustment was performed; this also showed the increase in macrolide resistant enterococci in the menthol group to be significant (*p <* 0.05).

**Figure 11:**
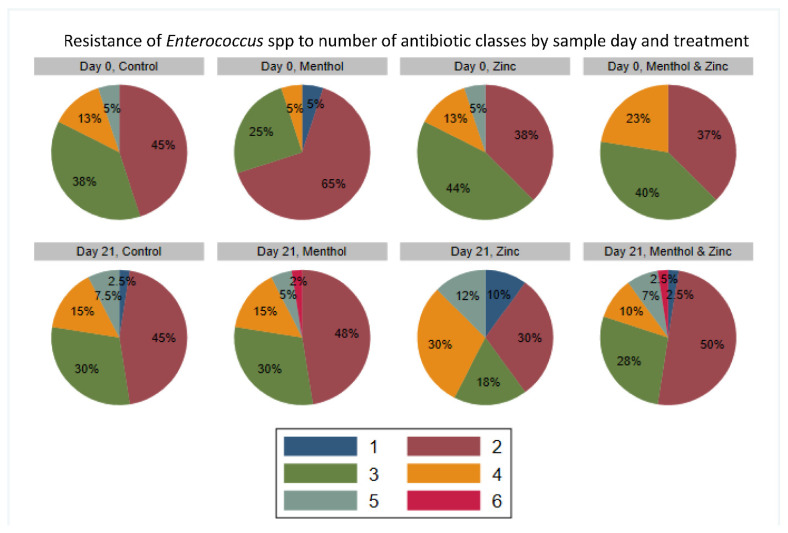
Resistance (%) of *Enterococcus* spp. isolates to number of antibiotic classes by sample day and treatment. Each color indicates the percentage of isolates resistant to the specified number of antibiotic classes (out of 13).

The percentage of isolates resistant to each of the distinct number of antibiotic classes by sample day and treatment (Figure 11) showed that all *Enterococcus* spp. isolates were resistant to at least one class of antibiotic; that is, with no pan-susceptible isolates observed. The menthol group showed an increase in the percentage of MDR isolates from 30% on Day 0 to 52% on Day 21. Correspondingly, the menthol group also showed an increase in the percentage of isolates resistant to four classes of antibiotics from 5% on Day 0 to 15% on Day 21. Additionally, the menthol group also had 2% of isolates resistant to six antibiotic classes on Day 21, compared to no isolates resistant to six classes on Day 0. The zinc group did not exhibit an increase in overall percentage of MDR isolates, with 62% of isolates resistant to three or more antibiotic classes on Day 0, and 60% resistant on Day 21. However, on Day 0, all isolates in the zinc group were resistant to at least two classes of antibiotic, while on Day 21, 10% of isolates were resistant to only one antibiotic class. The percentage of MDR isolates in the combined zinc and menthol group decreased from 63% on Day 0 to 47.5% on Day 21. The combined zinc menthol group also had 2.5% of isolates resistant to six antibiotic classes on Day 21, compared to none on Day 0. The MDR binary outcomes also were modeled using a multi-level mixed logistic regression.

**Figure 12:**
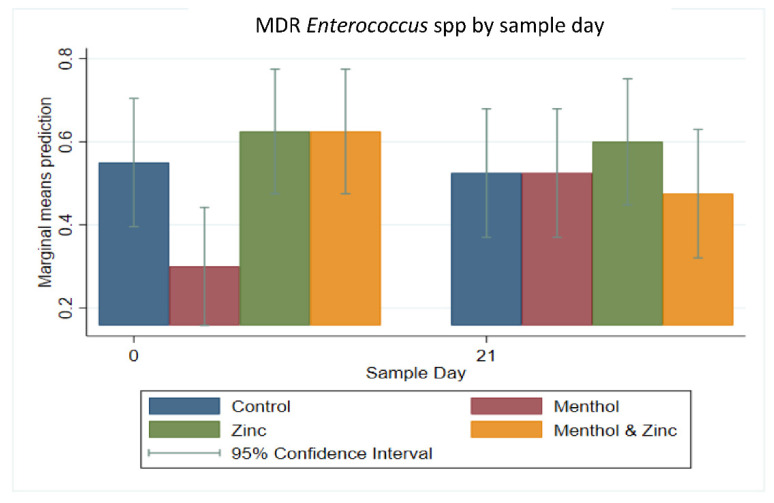
Marginal means with 95% confidence intervals of a 2 × 2 × 2 multi-level mixed logistic regression model, using factors of zinc, menthol and sample day on the binary outcome of MDR *Enterococcus* spp.

The results of a multi-level mixed effect logistic regression model on the binary outcome of MDR *Enterococcus* spp. isolates (Figure 12) showed that on Day 0 the menthol group had a significantly decreased predicted prevalence of MDR isolates compared to the zinc or zinc/menthol combined group (*p <* 0.05). Additionally, the predicted prevalence of MDR isolates from the combined zinc and menthol group decreased from Day 0 to Day 21; however, this decrease was not statistically significant (*p* > 0.05).

## 4. Discussion

This randomized controlled trial demonstrated that there were some trends towards increasing resistance, but few statistically significant effects of zinc or menthol supplementation on antibiotic resistance among fecal *E. coli*. The log_10_ CFU per gram of feces on plain MacConkey agar was significantly affected by sample day (i.e., study period) independent of treatment. Tetracycline resistance tended to increase for the combination zinc and menthol group on both quantified tetracycline-supplemented MacConkey agar and when directly assessing phenotypic resistance among randomly selected *E. coli* isolates. This was similar to results reported by Aperce et al. [30], who found that menthol significantly increased the prevalence of tetracycline-resistant *E. coli* in cattle. However, *Mentha piperita* (peppermint) essential oil and menthol have both been shown to inhibit quorum sensing, which regulates the expression of certain genes—including genes for antibiotic resistance—of Gram-negative organisms including *E. coli.* [35]. Quorum sensing also has been implicated in drug efflux pump regulation, including overexpression of the quorum sensing regulator *sdiA*, which can mediate multidrug resistance. Rahmati et al. [36] also found that *sdiA* null mutant *E. coli* were more sensitive to fluoroquinolones.

Our data showed no noticeable effects of sample day or treatment group pertaining to growth on ceftriaxone-supplemented MacConkey. Similarly, there was no effect on resistance of *E. coli* isolates to other cephem-class antibiotics, as determined using isolate-based analyses of isolates randomly selected from plain MacConkey agar. However, while zinc appeared to be associated with higher aminoglycoside and tetracycline resistance among *E. coli* isolates, this difference did not prove to be statistically significant (*p* > 0.05). This is in contrast to a previous study in pigs, in which heavy metals, particularly mercury, were associated with a decrease in aminoglycoside, tetracycline and cephalosporin resistance [37]. Additionally, among *E. coli* isolates the prevalence of multi-drug resistance (MDR) tended to increase, though not significantly, for the zinc and the combination zinc/menthol group. Interestingly, two previous studies suggest that high dietary zinc promotes MDR in pigs [38,39]. Yet another more recent study, also conducted in swine, cautioned against this observation and stated that tolerance to zinc was not associated with MDR [40]. At the time of our study, among beef cattle in the United States, there appears to be little evidence for co-selection of antibiotic resistance among fecal bacteria from animals fed supranutritional levels of zinc (300 ppm) or menthol fed at 0.3%.

Among enterococci, menthol was associated with significantly (*p <* 0.05) increased macrolide resistance among *Enterococcus* spp. isolates from Day 0 to Day 21. Additionally, the menthol group tended to be associated with an increase in the prevalence of MDR among isolates from Day 0 to Day 21. At the time of writing, there were few studies examining the effects of essential oils on antibiotic resistance, even though essential oils such as tea tree oil have been licensed for medicinal use in Australia since the 1920s [41]. When a variety of essential oils were screened for bactericidal activity, Shapiro et al. found that peppermint oil, which contains menthol, and tea tree oil were the most potent essential oils with action against obligate anaerobes and facultative anaerobes [42]. While tea tree oil has been licensed for the past 100 years, clinical resistance has yet to be reported [43]. However, after several generations of methicillin-resistant *Staphylococcus aureus* (MRSA) were exposed to tea tree oil, a subpopulation with increased MIC to tea tree oil emerged [44]. It is therefore plausible that after repeated exposure to menthol, in addition to antibiotic exposure, an MDR subpopulation of *Enterococcus* spp. could emerge. Conversely, after repeated exposure to oregano essential oil, *Serratia marcescens*, *Morganella morganii* and *Proteus mirabilis* exhibited a changed antibiotic resistance profile; however, this was not associated with an increased inhibitory concentration to oregano oil itself [45].

In our study, zinc was associated with increased erythromycin (macrolide) resistance, with significant increases in measured growth on erythromycin supplemented m-*Enterococcus* agar, and a significantly higher macrolide resistance among isolates from plain media analyzed using broth microdilution. These results are similar to a previous study by Hasman et al. [20], which showed supplementation of copper in piglets selected for the *tcr*B gene, which is strongly associated (due to co-location on a plasmid) with a gene (*ermB*) encoding resistance to macrolides. It should be noted, however, that the similarity is strictly between heavy metals and co-selection for macrolide resistance among enterococci, as the previous study used copper instead of zinc. Conversely, Jacob et al. [46] found that when cattle were fed a combination of zinc and copper, there were minimal effects on any associated increase in antibiotic resistance, and those authors did not find *tcrB* in the samples or among the enterococcal isolates.

## 5. Conclusions

Only temporal effects were significant among *E. coli*, while menthol was associated with decreased growth on tetracycline-supplemented agar. No significant treatment effects were present for *E. coli* among isolates. However, these trial data suggest that there are potential co-selection pressures occurring in populations of *Enterococcus* spp. when using supranutritional zinc and menthol as alternatives to antibiotics. No mechanistic explanations were pursued in this study. One limitation of this study relates to time constraints, since a longer period of supplementation with supranutritional zinc and menthol would have the potential to yield more sustained and significant effects. In all of the previous reported studies found in the literature, animals were supplemented for at least 28 days. By increasing the amount of time exposed to the alternatives, such as throughout the entire cattle feeding period of up to and greater than 180 days, further co-selection expanding resistance could occur. Longer and more definitive studies to further explore any associations are necessary, especially with menthol.

## Figures and Tables

**Table 1:** Antibiotics ordered by class, concentration ranges (mg/L) and interpretive breakpoints (for resistance) for the NARMS (National Antimicrobial Resistance Monitoring System) Gram-positive plate (CMV3AGP), using CLSI (Clinical and Laboratory Standards Institute) criteria and NARMS interpretive human breakpoints when a CLSI equivalent was unavailable.

Antibiotic	Class	Range	Breakpoint
Gentamicin	Aminoglycoside	128–1024	≥500
Kanamycin	Aminoglycoside	128–1024	≥1024
Streptomycin	Aminoglycoside	512–2048	>1000
Vancomycin	Glycopeptide	0.25–32	≥32
Tigecycline	Glycylcycline	0.015–0.5	≥0.5
Lincomycin	Lincosamide	1–8	≥8
Daptomycin	Lipopeptide	0.25–16	≥8
Erythromycin	Macrolide	0.25–8	≥8
Tylosin	Macrolide	0.25–32	≥32
Nitrofurantoin	Nitrofuran	2–64	≥128
Linezolid	Oxazolidinone	0.5–8	≥8
Penicillin	Penicillin	0.25–16	≥16
Chloramphenicol	Phenicol	2–32	≥32
Ciprofloxacin	Quinolone	0.12–4	≥4
Quinupristin/dalfopristin	Streptogramin	0.5–32	≥4
Tetracycline	Tetracycline	1–32	≥16

**Table 2:** Antibiotics ordered by class, concentration ranges (mg/L) and interpretive breakpoints (for resistance) for NARMS Gram-negative plate (CMV3AGNF), using CLSI criteria and NARMS interpretive human breakpoints when a CLSI equivalent was unavailable.

Antibiotic	Class	Range	Breakpoint
Gentamicin	Aminoglycoside	0.25–16	≥16
Streptomycin	Aminoglycoside	2–64	≥64
Cefoxitin	Cephem	0.5–32	≥32
Ceftiofur	Cephem	0.12–8	≥32
Ceftriaxone	Cephem	0.25–64	>4
Sulfisoxazole	Folate Pathway Inhibitor	16–256	≥512
Trimethoprim/sulfamethoxazole	Folate Pathway Inhibitor	0.12/2.38–4/76	≥4/76
Azithromycin	Macrolide	0.12–16	≥32
Ampicillin	Penicillin	1–32	≥32
Chloramphenicol	Phenicol	2–32	≥32
Ciprofloxacin	Quinolone	0.015–4	≥1
Nalidixic Acid	Quinolone	0.5–32	≥32
Tetracycline	Tetracycline	4–32	≥16
Amoxicillin/clavulanic acid	β-Lactam/β-Lactamase inhibitor combination	1/0.5–32/16	≥32/16

**Table 3:** Number and percentage (with 95% CI) of *E. coli* isolates that were resistant and the distribution of isolates (%) across the observed minimum inhibitory concentrations (MIC) for each antibiotic. Black vertical lines indicate the human medical interpretive breakpoint CLSI (or NARMS). Grey boxes indicate unmeasured values above and below highest and lowest limit of assayed antibiotic concentrations, respectively. Isolates which exceeded growth at the highest antibiotic concentration were placed into the next highest MIC column.

Antibiotic	Number Resistant (of 320 Tested)	% Resistant	95% Confidence Interval		MIC in µg/mL														
					0.02	0.03	0.06	0.13	0.25	0.50	1.00	2.00	4.00	8.00	16.00	32.00	64.00	128.00	256.00
Amoxicillin/Clavulanic Acid	3.00	0.94	0.19	2.72							4.69	18.75	60.94	14.38	0.31	0.31	0.63		
Ampicillin	42.00	13.30	9.63	17.32							5.31	42.50	37.50	1.56	0.00	13.13			
Azithromycin	2.00	0.63	0.08	2.24				0.00	0.00	0.94	0.94	8.13	78.75	10.31	0.31	0.63			
Cefoxitin	3.00	0.94	0.19	2.72						0.00	0.31	7.81	61.56	28.13	1.25	0.31	0.63		
Ceftiofur	3.00	0.94	0.19	2.72				2.81	21.25	73.75	0.94	0.31	0.00	0.63	0.31				
Ceftriaxone	3.00	0.94	0.19	2.72					98.13	0.94	0.00	0.00	0.31	0.00	0.31	0.00	0.31		
Chloramphenicol	48.00	15.00	11.27	19.39								2.50	46.25	35.63	0.63	0.63	14.37		
Ciprofloxacin	0.00	0.00	0.00	1.14 *	95.94	2.50	0.63	0.31	0.63	0.00	0.00	0.00	0.00						
Gentamicin	1.00	0.31	0.01	1.73					0.94	75.31	23.44	0.00	0.00	0.00	0.31				
Nalidixic Acid	3.00	0.94	0.19	2.72						0.31	4.69	76.25	17.81	0.00	0.00	0.31	0.63		
Streptomycin	73.00	22.84	18.33	27.81								0.00	16.56	50.94	4.06	5.63	11.56	11.25	
Sulfisoxazole	67.00	20.94	16.61	25.81											73.13	5.00	0.63	0.31	20.94
Tetracycline	148.00	46.25	40.69	51.88									44.38	9.38	2.50	4.06	39.69		
Trimethoprim/sulfamethoxazole	2.00	0.63	0.08	2.24				87.81	7.50	3.75	0.31	0.00	0.63						

* 97.5% One-sided CI.

**Table 4:** Percentage of *Enterococcus* spp. isolates that were resistant and their distribution across minimum inhibitory concentrations (MIC) for each antibiotic. Black vertical lines indicate the human CLSI (or NARMS) interpretive breakpoint, grey boxes indicate areas above and below highest and lowest limit of assay antibiotic concentrations, respectively. Isolates which exceeded growth at the highest antibiotic concentration were placed in the next MIC column.

Antibiotic	Number Resistant (of 320 Tested)	% Resistant	95% Confidence Interval		MIC in µg/mL														
					0.02	0.03	0.06	0.13	0.25	0.5	1	2	4	8	16	32	64	128	256
Chloramphenicol	2.00	0.63	0.08	2.24								0.31	13.80	80.00	5.31	0.63			
Ciprofloxacin	18.00	5.63	3.37	8.74					0.31	17.80	43.40	32.80	4.69	0.94					
Daptomycin	28.00	8.75	5.89	12.40					1.25	0.31	2.50	20.60	66.60	8.75	0.00				
Erythromycin	57.00	17.81	13.80	22.50					50.30	7.81	2.50	8.13	13.40	3.75	14.10				
Gentamicin	0.00	0.00	0.00	1.15 *														100.00	0.00
Kanamycin	3.00	0.94	0.19	2.72														91.90	6.56
Lincomycin	292.00	91.25	87.60	94.10							8.44	0.31	0.00	5.31	85.90				
Linezolid	1.00	0.31	0.01	1.73						0.31	6.88	68.10	24.40	0.31					
Nitrofurantoin	52.00	16.25	12.40	20.80								0.00	0.00	2.19	2.18	16.30	62.50	16.30	
Penicillin	1.00	0.31	0.01	1.73					2.50	7.19	12.20	15.60	54.40	7.81	0.31				
Streptomycin	3.00	0.94	0.19	2.72															
Quinupristin/Dalfopristin	106.00	33.13	28.00	38.60						10.00	1.56	55.30	32.80	0.31	0.00	0.00			
Tetracycline	106.00	33.13	28.00	38.60							64.10	1.25	0.00	1.56	2.50	3.75	26.90		
Tigecycline	0.00	0.00	0.00	1.15 *	0.00	11.30	61.90	26.90	0.00	0.00									
Tylosin	59.00	18.44	14.30	23.10					0.00	0.00	1.25	16.90	20.30	37.50	5.63	0.94	17.50		

* 97.5% One-sided CI.

## Data Availability

The datasets used and/or analyzed are available from the corresponding author upon reasonable request.
